# Photoinactivation of catalase sensitizes a wide range of bacteria to ROS-producing agents and immune cells

**DOI:** 10.1172/jci.insight.153079

**Published:** 2022-05-23

**Authors:** Pu-Ting Dong, Sebastian Jusuf, Jie Hui, Yuewei Zhan, Yifan Zhu, George Y. Liu, Ji-Xin Cheng

**Affiliations:** 1 Department of Biomedical Engineeriˆng,; 2 Photonics Center, and; 3 Department of Chemistry, Boston University, Boston, Massachusetts, USA.; 4 Division of Pediatric Infectious Diseases and Research Division of Immunology, Department of Biomedical Sciences, Cedars-Sinai Medical Center, Los Angeles, California, USA.; 5 Division of Infectious Diseases, Department of Pediatrics, UCSD, San Diego, California, USA.

**Keywords:** Infectious disease, Microbiology, Bacterial infections

## Abstract

Bacteria have evolved to cope with the detrimental effects of ROS using their essential molecular components. Catalase, a heme-containing tetramer protein expressed universally in most aerobic bacteria, plays an indispensable role in scavenging excess hydrogen peroxide (H_2_O_2_). Here, through use of wild-type and catalase-deficient mutants, we identified catalase as an endogenous therapeutic target of 400–420 nm blue light. Catalase residing inside bacteria could be effectively inactivated by blue light, subsequently rendering the pathogens extremely vulnerable to H_2_O_2_ and H_2_O_2_-producing agents. As a result, photoinactivation of catalase and H_2_O_2_ synergistically eliminated a wide range of catalase-positive planktonic bacteria and *P*. *aeruginosa* inside biofilms. In addition, photoinactivation of catalase was shown to facilitate macrophage defense against intracellular pathogens. The antimicrobial efficacy of catalase photoinactivation was validated using a *Pseudomonas aeruginosa–*induced mouse abrasion model. Taken together, our findings offer a catalase-targeting phototherapy approach against multidrug-resistant bacterial infections.

## Graphical abstract

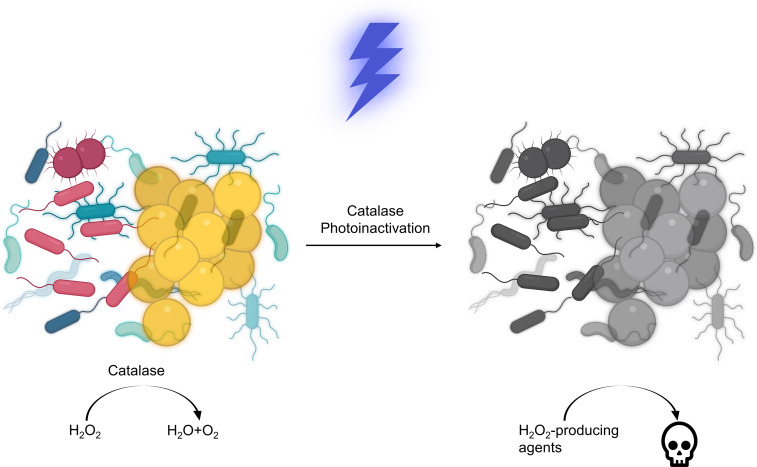

## Introduction

Antibiotic resistance has remained one of the biggest threats to global health over the past decades. In United States alone, it is estimated that at least 2.8 million people are infected by antibiotic-resistant bacterial infections annually (1). Despite this alarming number, the pace of antibiotic resistance development is faster than that of clinical introduction of new antibiotics (2). If no efforts are made to curtail this situation, loss of life from antibiotic-resistant infections might surpass that from cancer, and 10 million people will be killed worldwide by 2050 (3). Moreover, the imprudent use of antibiotics from clinical overprescription and by the food industry escalates the selection of multidrug-resistant or even pan-drug-resistant bacteria (4).

Confronted with this dire situation, antimicrobial blue light has emerged as a novel approach to combat multidrug-resistant bacterial infections (5, 6). Blue light in the 405 to 420 nm or the 450 to 470 nm optical windows has demonstrated bactericidal effects on a wide range of microbial species, including Gram-positive bacteria, Gram-negative bacteria, mycobacteria, and molds (6). Of note, blue light has been utilized for clinical treatment of *Propionibacterium acnes* (7). *Helicobacter pylori*, the major cause of peptic ulcer disease, can efficiently inactivated in vitro by visible light (8, 9). Besides planktonic-form bacteria, blue light also decreased the viability of *Pseudomonas aeruginosa* (*P*. *aeruginosa*), methicillin-resistant *Staphylococcus aureus* (MRSA) USA300, and *Candida albicans* (*C. albicans*) in biofilm conditions (10). Importantly, no evidence of blue light resistance development by pathogens has been documented after consecutive blue light treatments (11–13). Blue light has also been allied with other antimicrobial agents to eradicate bacteria. For example, quinine in combination with blue light exposure has shown efficacy to eliminate Gram-negative *P*. *aeruginosa*, *Acinetobacter baumannii* (*A*. *baumannii*; ref. 5), and *C. albicans* (14). Blue light irradiance was also reported to enhance the inactivation efficacy of low-concentration chlorinated disinfectants toward *Clostridium difficile* (15). Blue light (460 nm) plus hydrogen peroxide (H_2_O_2_) exhibited high efficacy to eradicate MRSA by devastating its functional membrane domain (16, 17).

Despite these advances, the working mechanism of antimicrobial blue light has remained elusive for years. Endogenous metal-free porphyrin and riboflavin has been considered as the major molecular targets (6). It is assumed that ROS produced from photodynamic reaction between blue light and these endogenous chromophores leads to bacterial death. However, this hypothesis has remained controversial. The concentration of endogenous porphyrins or riboflavin is as low as 2 to 4 × 10^–3^ mg/mL (18), and a precursor, *δ*-aminolevulinic acid (ALA), was routinely administered to enhance the intracellular production of porphyrins when treating *E*. *coli* and other bacteria (19) by 400–420 nm blue light. In the absence of ALA, 407–420 nm blue light with a dose of 50 J/cm^2^ did not exhibit significant bactericidal effects on *Staphylococcus aureus* (*S*. *aureus*), *A*. *baumannii*, and *E*. *coli* (19). Also, it has been reported that the total amount of coproporphyrins was not a contributing factor of the antimicrobial efficacy of blue light treatment (20). Alternatively, pyocyanin, the prototypical green pigment produced by *P*. *aeruginosa* (21), has been suggested to serve as a photosensitizer upon blue light exposure (22, 23). Pyoverdine, a naturally occurring fluorescent pigment in *P*. *aeruginosa*, was also believed to undergo photodynamic reactions upon absorption of 415 nm light (24).

Very recently, our studies found that blue light at 460 nm is able to bleach staphyloxanthin (16), a ROS scavenger as well as an endogenous golden pigment residing in *S*. *aureus* functional membrane domains (25, 26), making this pathogen vulnerable to low-concentration H_2_O_2_ (16). Follow-up studies using pulsed blue light have shown more effective capability of photobleaching of staphyloxanthin, which sensitizes *S*. *aureus* to a broad spectrum of antibiotics (17) and to silver nanoparticles (27). In an independent study, it was shown that photobleaching of another ROS scavenger and pigment, granadaene, by 430 nm light is able to reduce the virulence and increase the antimicrobial susceptibility of *Streptococcus agalactiae* (28). Collectively, these findings suggest an alternative working mechanism of antimicrobial blue light, which is based on photoinactivation of intrinsic ROS-scavenging molecules inside the pathogen.

It is well established that aerobic microorganisms produce ROS endogenously when flavin, quinol, or iron cofactors are autoxidized in the processes of cellular metabolism and respiration (29). When bacteria are challenged with antibiotics or other stressors, a cascade of ROS can be generated (30, 31). Excess ROS damage DNA, certain metalloproteins, lipids, and other essential cellular components (32). To scavenge excess ROS and maintain the intracellular homeostasis, bacteria have evolved to be armed with an array of strategies. Of these, catalase, an enzyme with a turnover of 2.8 × 10^6^ molecules per second (33), very efficiently converts H_2_O_2_ into O_2_ and water. For this reason, visualization of oxygen bubbles in the presence of H_2_O_2_ and Triton X-100 offers a simple method to quantify catalase activity (34). In the absence of catalase, Fenton reaction between H_2_O_2_ and iron produces various radicals, such as HO· and HOO·, and poses lethal threats to bacteria (35). Importantly, it was shown as early as 1965 that catalase could be inactivated by visible light (36), with the optical density at 405 nm (the primary absorption peak) diminishing as the irradiance continued. The underlying mechanism was presumed to be due to the dissociation of prosthetic heme groups from the tetramer protein (37). Nonetheless, whether photoinactivation of catalase could be harnessed to eliminate pathogenic bacteria is yet to be explored.

Here, we show that catalase expressed universally in common pathogens is a key target of antimicrobial blue light in the 400 to 420 nm optical window. Blue light illumination inactivated catalase by destroying the porphyrin rings. Using the same dose, nanosecond (ns) pulsed blue light at 410 nm induced more effective inactivation of catalase than the continuous-wave (CW) 410 nm irradiance. We further demonstrate that photoinactivation of catalase sensitized bacterial pathogens, both in planktonic form and in biofilms, to exogenous, nonbactericidal, low-concentration H_2_O_2_. Moreover, photoinactivation of catalase sensitized pathogens to certain antibiotics that exert their lethal effects on bacteria partly through ROS-induced physiological alterations (38). Photoinactivation of catalase enhanced immune cell elimination of intracellular MRSA USA300 and *P*. *aeruginosa*. In a *P*. *aeruginosa*–induced mouse skin abrasion model, photoinactivation of catalase effectively reduced the pathogen burden. Taken together, our findings offer a new way to combat multidrug-resistant bacterial infections.

## Results

### Bacterial catalase can be inactivated by blue light irradiance.

**Figure 1:**
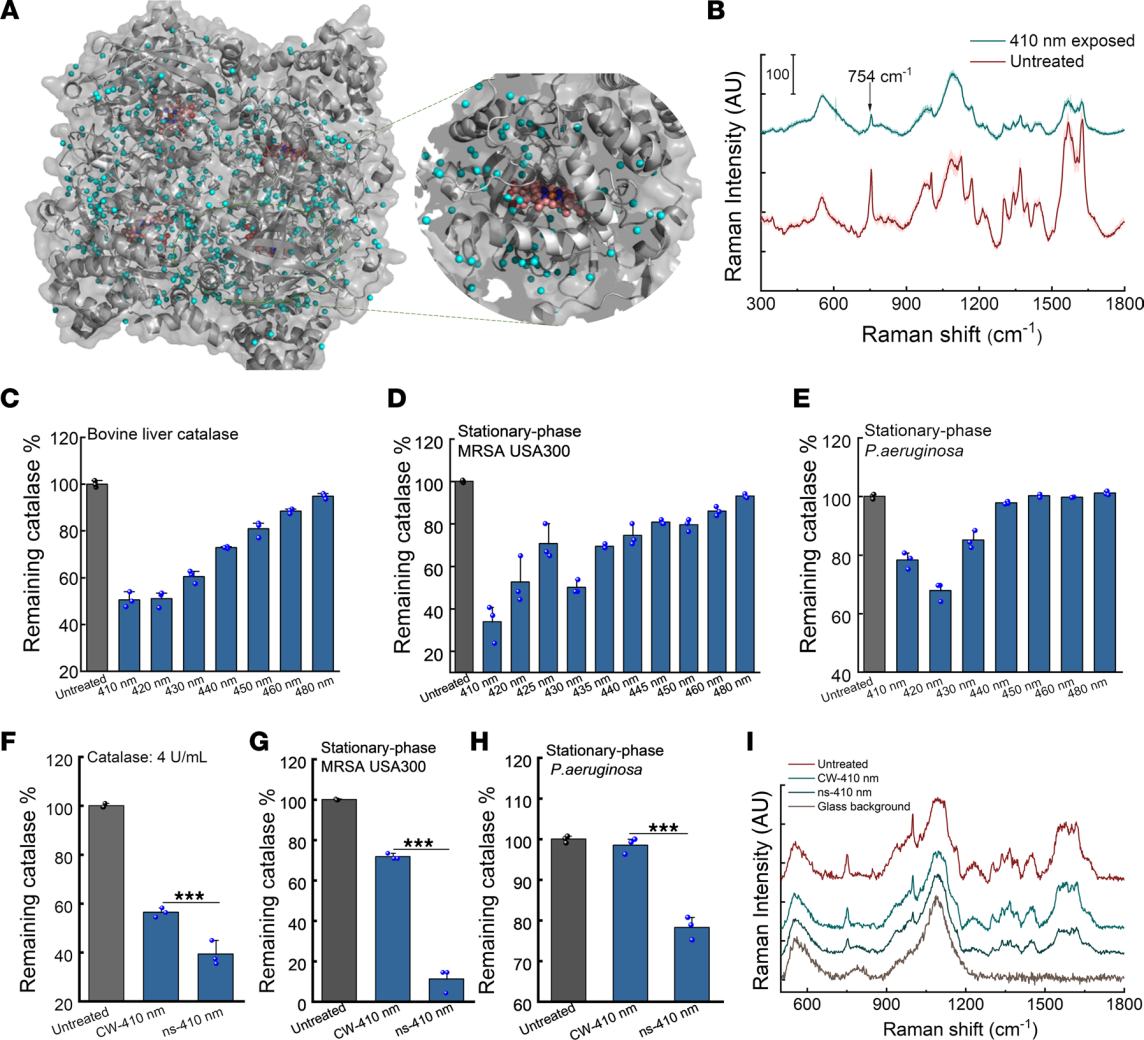
Catalase inactivation by CW and pulsed blue light in a wavelength-dependent manner. (**A**) *P*. *aeruginosa* catalase structure through PyMOL simulation. PDB 4E37. (**B**) Raman spectra of a bovine liver catalase film dried on a cover slide before (untreated) and after 410 nm light exposure. The Raman peak at 754 cm^–1^ is highlighted by an arrow. 410 nm: 50 mW/cm^2^, 10 minutes. SDs at each Raman shift are shaded. (**C**–**E**) Remaining catalase percentage of bovine liver catalase (**C**, 2.5 U/mL), stationary-phase MRSA USA300 (**D**), and stationary-phase *P*. *aeruginosa* PAO1 (**E**) under blue light exposure at different wavelengths. (**F**–**H**) Comparison of CW and ns-410 nm exposure on inhibiting bovine liver catalase (**F**, 4 U/mL), catalase from stationary-phase MRSA USA300 (**G**) and stationary-phase *P*. *aeruginosa* PAO1 (**H**). (**I**) Raman spectra of bovine liver catalase film dried on a cover slide under CW-410 and ns-410 exposure. Light: 50 mW/cm^2^, 5 minutes. Data: Mean + SD from 3 replicates for panels **B**–**H**. Statistical analysis was obtained through Student’s unpaired 2-tailed *t* test. ***P < 0.001.

Catalase is a tetramer of 60,000-dalton subunits, containing 4 prosthetic heme rings per tetramer. As shown in Figure 1A, the porphyrin rings stay deep inside the hydrophobic pocket of catalase from *P*. *aeruginosa*. Since bovine liver catalase was shown to be inactivated by visible light decades ago (36) but with an unclear underlying mechanism, we asked whether and how catalase structure changes in the presence of light exposure near its absorption around 405 nm. For this purpose, Raman spectra of a dried bovine liver catalase film were obtained before and after 410 nm exposure. Catalase exhibits its prototypical peaks at 754 cm^–1^ and 1200 cm^–1^ to 1500 cm^–1^ because of porphyrin rings (Figure 1B and ref. 39). Interestingly, the Raman intensity at those peaks drastically dropped after 410 nm exposure, indicating a possible dissociation of the heme ring from catalase.

To further understand the impact of 410 nm exposure on catalase, transient absorption microscopy was utilized to record the time-resolved photobleaching behavior of bovine liver catalase and of catalase inside MRSA USA300, *P*. *aeruginosa*, and *Salmonella* *enterica* (*S.* *enterica*; Supplemental Figure 1; supplemental material available online with this article; https://doi.org/10.1172/jci.insight.153079DS1). Samples were excited by a pump beam at wavelength of 410 nm, and transient absorption signals were detected by a probe beam at a wavelength of 520 nm. As shown in Supplemental Figure 1, the transient absorption signal from bovine liver catalase decayed as the irradiance continued (Supplemental Figure 1, A–C). Moreover, this decay (Supplemental Figure 1D) followed a second-order photobleaching model (16), suggesting an interaction between heme rings within the tetramer. Consistently, photoinactivation of catalase from MRSA USA300 (Supplemental Figure 1E), *P*. *aeruginosa* (Supplemental Figure 1F), and *S.* *enterica* (Supplemental Figure 1G) followed a similar photobleaching trend as that of bovine liver catalase. Functionally, after 410 nm exposure, no apparent bubbles were observed when adding 0.3% H_2_O_2_ to the light-exposed catalase solution (Supplemental Video 1). In summary, the enzyme-bound heme rings could be dissociated from the protein upon 410 nm irradiance, causing the protein to malfunction.

Next, we asked what the optimal wavelength to inactivate catalase is. To investigate this, we used an OPO laser (Opolette 355, OPOTEK) and varied the laser wavelength under the same power density and dose. A catalase kit that quantifies the residual H_2_O_2_ was then used to indirectly measure the remaining catalase percentage to evaluate the efficacy of light exposure. As shown in Figure 1C, 410 to 420 nm demonstrated the highest efficiency to inactivate bovine liver catalase (~50% inactivation at a dose of 15 J/cm^2^). Catalase plays a dominant role in conversion of H_2_O_2_ into water and O_2_ in bacteria (40). We adopted the same protocol to evaluate the remaining catalase percentage in stationary-phase MRSA USA300 (Figure 1D) and *P*. *aeruginosa* (Figure 1E). Consistently, 410 to 420 nm exposure most effectively attenuated the bacterial catalase activity. Therefore, 410 nm was utilized for catalase inactivation for the subsequent experiments.

Since photoinactivation of catalase is likely due to the dissociation of the heme rings from the protein following a second-order photobleaching model, we asked whether high-peak power pulsed (e.g., nanosecond) 410 nm (ns-410) blue light more effectively inactivates catalase compared with the 410 nm continuous wave (CW-410). To answer this question, 3 experiments were conducted. First, we used the same catalase kit to quantify the H_2_O_2_ conversion efficacy of bovine liver catalase (Figure 1F), stationary-phase MRSA USA300 (Figure 1G), and stationary-phase *P*. *aeruginosa* (Figure 1H) after ns-410 and CW-410 blue light exposure, respectively. Our data repeatedly exhibited the pattern that ns-410 was significantly more effective for catalase inactivation compared with CW-410 (*P* < 0.001). Second, Raman spectra of bovine liver catalase film dried on a glass substrate were obtained after ns-410 and CW-410 exposure. Of note, under the same dose, ns-410 nm displayed a better capability to bleach catalase as Raman peaks at 754 cm^–1^ and 1200 to 1500 cm^–1^ drastically dropped (Figure 1I). Third, we compared the heights of oxygen bubble foams catalase produced in the presence of H_2_O_2_ and Triton X-100 after ns-410 and CW-410 exposure at the same dose. As shown in Supplemental Figure 2, ns-410–treated groups indeed showed much lower foam heights. Collectively, ns-410 exposure was more effective to inactivate catalase compared with CW-410. Notably, ns-410 exposure (10 Hz) also alleviated the heat accumulation issue that is generally concomitant with longtime CW light exposure.

### Photoinactivation of catalase sensitizes pathogenic bacteria to low H_2_O_2_ concentrations.

**Figure 2:**
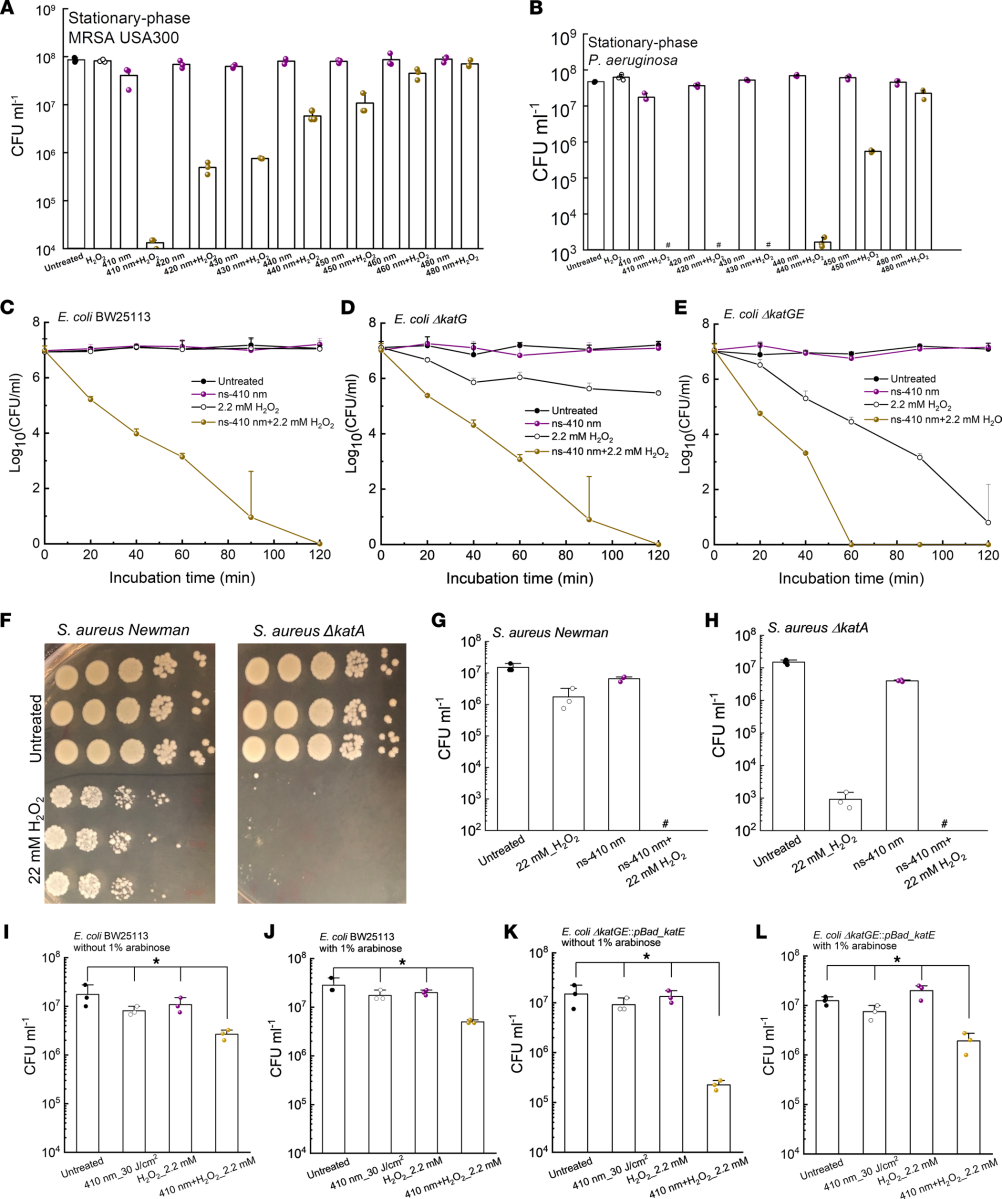
Photoinactivation of catalase effectively sensitizes pathogenic bacteria to H_2_O_2_. (**A**) CFU/mL of stationary-phase MRSA USA300 under various treatments. Dose: 50 mW/cm^2^, 5 minutes. H_2_O_2_: 22 mM, 30-minute incubation time. (**B**) CFU/mL of stationary-phase *P*. *aeruginosa* PAO1 under various treatments. Dose: 50 mW/cm^2^, 5 minutes. H_2_O_2_: 22 mM, 30-minute incubation time. (**C**–**E**) Time-killing curves of wild-type *E*. *coli* BW25113 (**C**), *E*. *coli ΔkatG* (**D**), and *E*. *coli ΔkatGE* (**E**) under different treatment schemes. ns-410 nm: 30 mW, 8 minutes, 14 J/cm^2^. (**F**) CFU plates of *S*. *aureus Newman* along with its isogenic catalase-mutant *S*. *aureus ΔkatA* with/without H_2_O_2_ treatment. (**G** and **H**) CFU/mL of *S*. *aureus Newman* (**G**) and *S*. *aureus ΔkatA* (**H**) under different treatment schemes. (**I**–**L**) Viable *E*. *coli* BW25113 (**I** and **J**) and *E*. *coli* *ΔkatGE*:*pBad_katE* (**K** and **L**) were enumerated after incubating with/without H_2_O_2_ (2.2 mM) for 30 minutes in the absence/presence of arabinose (4 hours) after 3 J/cm^2^ 410 nm treatment. Data: Mean + SD from at least 3 biological replicates for all panels. Pound sign indicates the CFU results are below the detection limit. Statistical analysis was obtained by Student’s 2-tailed unpaired *t* test (compared with the untreated group) and 1-way ANOVA. *P < 0.05.

Since the primary role of catalase is to degrade H_2_O_2_ into water and oxygen, we then investigated whether photoinactivation of catalase renders bacteria vulnerable to H_2_O_2_. We tested the viability of MRSA USA300 and *P*. *aeruginosa* after different treatments (various light wavelengths, light doses, and H_2_O_2_ concentrations) by colony-forming units (CFU/mL) enumeration. H_2_O_2_ at 22 mM (or 0.075%) did not show apparent bactericidal effect (Figure 2, A and B). Yet, 4-log_10_ reduction was achieved in the 410 nm plus H_2_O_2_ treated group, indicating a very strong synergy between 410 nm exposure and H_2_O_2_ for bacterial eradication.

It is noteworthy that the more recalcitrant stationary-phase *P*. *aeruginosa* PAO1 was completely eradicated by 410 nm plus H_2_O_2_ (Figure 2B). When tuning the irradiance wavelengths, the killing pattern was similar to that of catalase inactivation (Figure 1, C–E), suggesting catalase was a key target of blue light irradiance. In addition, ns-410 plus H_2_O_2_ treatment of MRSA USA300 outperformed CW-410 plus H_2_O_2_ treatment by approximately 3-log_10_ (Supplemental Figure 3A). The combination treatment that made use of ns-410 achieved total eradication of *P*. *aeruginosa* PAO1 and outperformed the CW-410 plus H_2_O_2_ treatment (Supplemental Figure 3B). These findings are in line with ns-410 being more efficient at inactivating catalase than CW-410 when using the same dose.

To further investigate whether it is catalase that primarily accounts for the synergy between 410 nm exposure and H_2_O_2_, we measured the viability of *E*. *coli* BW25113 along with its catalase-deficient mutants, *E*. *coli* *ΔkatG* (single mutant) and *E*. *coli* *ΔkatGE* (double mutant) under the same treatments. First, a time-killing CFU assay was conducted. Complete eradication of *E*. *coli* BW25113 was obtained in the ns-410 plus H_2_O_2_ treated group after 2 hours of incubation in PBS whereas 410 nm alone or H_2_O_2_ alone barely killed the bacteria (Figure 2C). The synergistic activity of a combination treatment is defined as a >2-log_10_ decrease in the CFU/mL compared with that obtained with the most active agent alone (41). Therefore, there is an effective synergy between 410 nm blue light and H_2_O_2_.

Catalase-deficient *E*. *coli* *ΔkatG* and *E*. *coli* *ΔkatGE* had baseline susceptibility to H_2_O_2_ drastically higher than the isogenic wild-type *E*. *coli* (Figure 2, D and E). The double-mutant *E*. *coli* *ΔkatGE* exhibited no visible oxygen bubble formation with the addition of H_2_O_2_ (Supplemental Figure 4). Moreover, *E*. *coli* *ΔkatGE* exhibited similar susceptibility to H_2_O_2_ killing compared to wild-type *E*. *coli* BW25113 exposed to ns-410 plus H_2_O_2_ in a time-kill assay, corroborating that catalase was the primary target of 410 nm light. More effective killing of the catalase-deficient mutants by 410 nm plus H_2_O_2_ was observed when compared with H_2_O_2_ treatment alone, suggesting the existence of additional molecular targets for 410 nm exposure.

To confirm that catalase is the primary target of blue light and inactivation of its underlying function accounts for the synergy between 410 nm and H_2_O_2_, we further applied the treatments to *S*. *aureus Newman* along with its isogenic catalase-deficient mutant *S*. *aureus* *ΔkatA*. At a concentration of 22 mM, H_2_O_2_ eliminated less than 1-log_10_ of *S*. *aureus* *Newman* yet reduced around 4-log_10_ of *S*. *aureus* *ΔkatA* (Figure 2F). When combining 410 nm exposure and H_2_O_2_, total eradications were obtained for both *S*. *aureus Newman* (Figure 2G) and *S*. *aureus* *ΔkatA* (Figure 2H).

To consolidate the catalase inactivation hypothesis, we designed a rescue experiment in which catalase-deficient *E*. *coli* *ΔkatGE* was transformed with a pBad-HPII plasmid (Addgene 105839) to form a rescue strain, *E*. *coli* *ΔkatGE*:*pBad_katE*. Briefly, catalase expression of *E*. *coli* *ΔkatGE*:*pBad_katE* could be promoted in the presence of arabinose. After transformation, both wild-type *E*. *coli* BW25113 and *E*. *coli* *ΔkatGE*:*pBad_katE* were cultured to log phase. Then 30 J/cm^2^ 410 nm blue light was applied to treat *E*. *coli* BW25113 along with *E*. *coli* *ΔkatGE*:*pBad_katE*. Bacteria were then collected and incubated inside M9 media supplemented with 1% arabinose for 4 hours at 37°C. Subsequently, 2.2 mM H_2_O_2_ was added to the bacterial suspension, followed by CFU assay to quantify viable bacterial cells.

As shown in Figure 2, I–L, the addition of arabinose had negligible effects on the CFU reduction of 410 nm plus H_2_O_2_-treated wild-type *E*. *coli* BW25113 under the applied treatment scenario (Figure 2, I and J). Interestingly, in the case of *E*. *coli* *ΔkatGE*:*pBad_katE*, with the presence of arabinose, there was only 1-log_10_ reduction of CFU/mL in the 410 nm plus H_2_O_2_ treated group (Figure 2L), whereas significantly more killing, 2-log_10_ reduction of CFU/mL, showed up in the 410 nm plus H_2_O_2_ treated group when promoter arabinose was absent (Figure 2K).

These data collectively demonstrate that catalase gene complementation can rescue catalase-deficient *E*. *coli* *ΔkatGE* in the presence of H_2_O_2_ stress and further suggest that catalase is a major molecular target of 410 nm light exposure.

### Catalase photoinactivation and H_2_O_2_ synergistically eradicate a wide range of bacteria.

Having established the synergy between 410 nm exposure and H_2_O_2_ in eradication of *E*. *coli*, MRSA USA300, and *P*. *aeruginosa*, we next asked whether this synergy works on other pathogenic bacteria. In 2017, WHO published a list of 12 global priority pathogens, including *A*. *baumannii*, *P*. *aeruginosa*, and *Salmonellae* (42), most of which are catalase positive. It was reported that *A*. *baumannii* can induce severe mucous membrane infections or even bacteremia (43). *P*. *aeruginosa* has been the main culprit of infection among patients with burn wounds, cystic fibrosis, acute leukemia, an organ transplants (44). *Salmonellae*, the most commonly isolated foodborne pathogens, lead to approximately 3 million deaths worldwide annually due to *Salmonella* gastroenteritis (45). Therefore, we explored whether 410 nm exposure and H_2_O_2_ are effective against these life-threatening pathogens.

**Figure 3:**
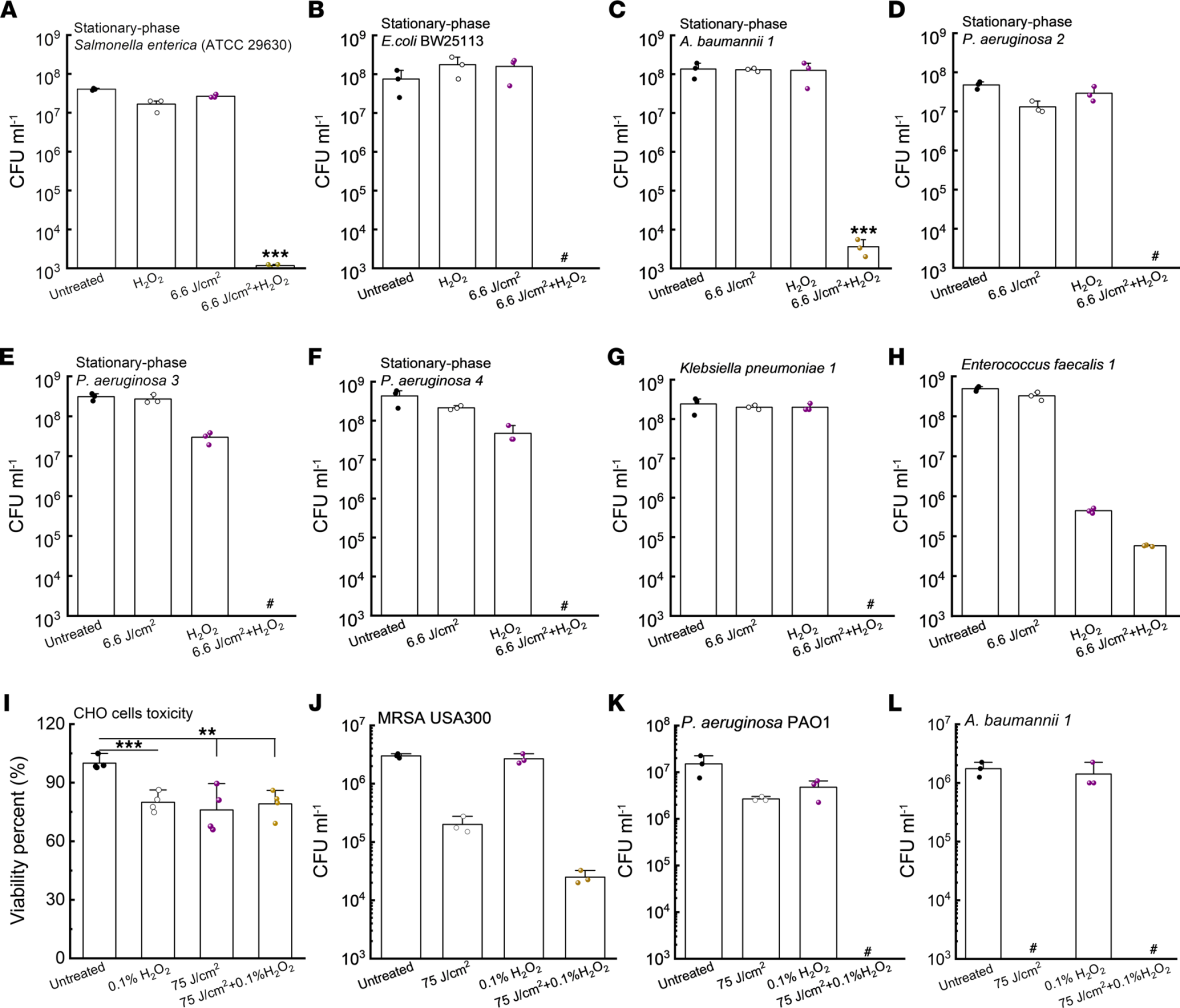
Photoinactivation of catalase sensitizes a wide range of pathogenic bacteria to H_2_O_2_. (**A**–**H**) CFU/mL of stationary-phase *S*. *enterica* ATCC 29630 (**A**), stationary-phase *E*. *coli* BW25113 (**B**), stationary-phase *A*. *baumannii 1* (**C**), stationary-phase *P*. *aeruginosa* strains (**D**–**F**), *Klebsiella pneumonia 1* (**G**), and *Enterococcus faecalis 1* (**H**) under various treatment schemes. 410 nm exposure: 6.6 J/cm^2^, H_2_O_2_: 22 mM, 30-minute culture time (for **A**–**H**). (**I**) Toxicity test of CHO cells by MTT assay under different treatment schemes. (**J**–**L**) CFU/mL of log-phase MRSA USA300 (**J**), log-phase *P*. *aeruginosa* PAO1 (**K**), and log-phase *A*. *baumannii 1* (**L**) under different treatment schemes. 410 nm exposure: 75 J/cm^2^, H_2_O_2_: 0.1%. Serial dilution or MTT assay was conducted after 1-minute incubation time (for **I**–**L**). Data: Mean + SD from 3 replicates for all panels. Pound sign indicates the CFU results are below the detection limit. Significant difference was determined by Student’s 2-tailed unpaired *t* test and 1-way ANOVA. Significant difference from all the other groups unless noted. ***P < 0.001; **P < 0.01.

Consistently, we found that total eradications (around 8-log_10_ reduction) were achieved when the treatment was applied to stationary-phase *S. enterica* ATCC 29630 (Figure 3A) and stationary-phase *E*. *coli* BW25113 (Figure 3B). A 5-log_10_ reduction of stationary-phase *A*. *baumannii 1* (Figure 3C) was achieved in the 410 nm plus H_2_O_2_ treated group. Notably, this synergistic cocktail therapy achieved total eradication (around 8-log_10_ reduction) of multiple *P*. *aeruginosa* strains (Figure 3, D–F) and *Klebsiella pneumonia 1* strains (Figure 3G). We then asked if this synergy still holds for catalase-negative pathogens, e.g., *Enterococcus faecalis* (*E*. *faecalis 1*; ref. 46). As shown in Figure 3H, H2O_2_ alone effectively killed *E*. *faecalis 1* by around 3-log_10_, but the enhanced bactericidal effect associated with 410 nm exposure was not as significant as that for catalase-positive pathogens. Taken together, 410 nm plus H_2_O_2_ can effectively eradicate wide-ranging life-threatening pathogens.

To query the translational potential of this synergistic therapy, we inquired whether short H_2_O_2_ (with higher concentration) incubation time remains effective at eliminating bacteria after 410 nm exposure. Prior to that, we evaluated the toxicity of 0.1% (w/v) H_2_O_2_ (30 mM, 2-minute incubation), 410 nm exposure (75 J/cm^2^), and 410 nm exposure plus 0.1% H_2_O_2_ to a CHO cell line through MTT assay. A 0.1%~0.2% concentration of H_2_O_2_ was chosen since it was reported that H_2_O_2_ in commercial disinfectant formulations is in the range of 0.1% to 3% (47). As shown in Figure 3I, viability of CHO cells (around 80%) treated with 410 nm (75 J/cm^2^) plus H_2_O_2_ (0.1%, 1-minute incubation) was similar to CHO cells treated with 410 nm or H_2_O_2_.

Hence, we moved next to interrogate the bactericidal effect of short treatment against log-phase MRSA USA30, *P*. *aeruginosa* PAO1, and *A*. *baumannii 1*. Enhanced killing of log-phase MRSA USA300 was observed with 410 nm plus H_2_O_2_ treatment compared with treatment with H_2_O_2_ or 410 nm alone (Figure 3J). Total eradication was achieved in the case of log-phase *P*. *aeruginosa* PAO1 (Figure 3K) and log-phase *A*. *baumannii 1* (Figure 3L) with 410 nm and 1-minute 0.1% to 0.2% H_2_O_2_. Log-phase MRSA USA300, *P*. *aeruginosa* PAO1, and *A*. *baumannii 1* were particularly sensitive to 410 nm exposure (Figure 3L) compared with the bacteria grown to stationary phase, indicating possible variation in catalase expression with growth condition. Collectively, 410 nm plus short exposure to H_2_O_2_ are sufficient to achieve substantial bacterial reduction with negligible toxicity.

### Photoinactivation of catalase enhances microbicidal activity of H_2_O_2_-producing antibiotics.

**Figure 4:**
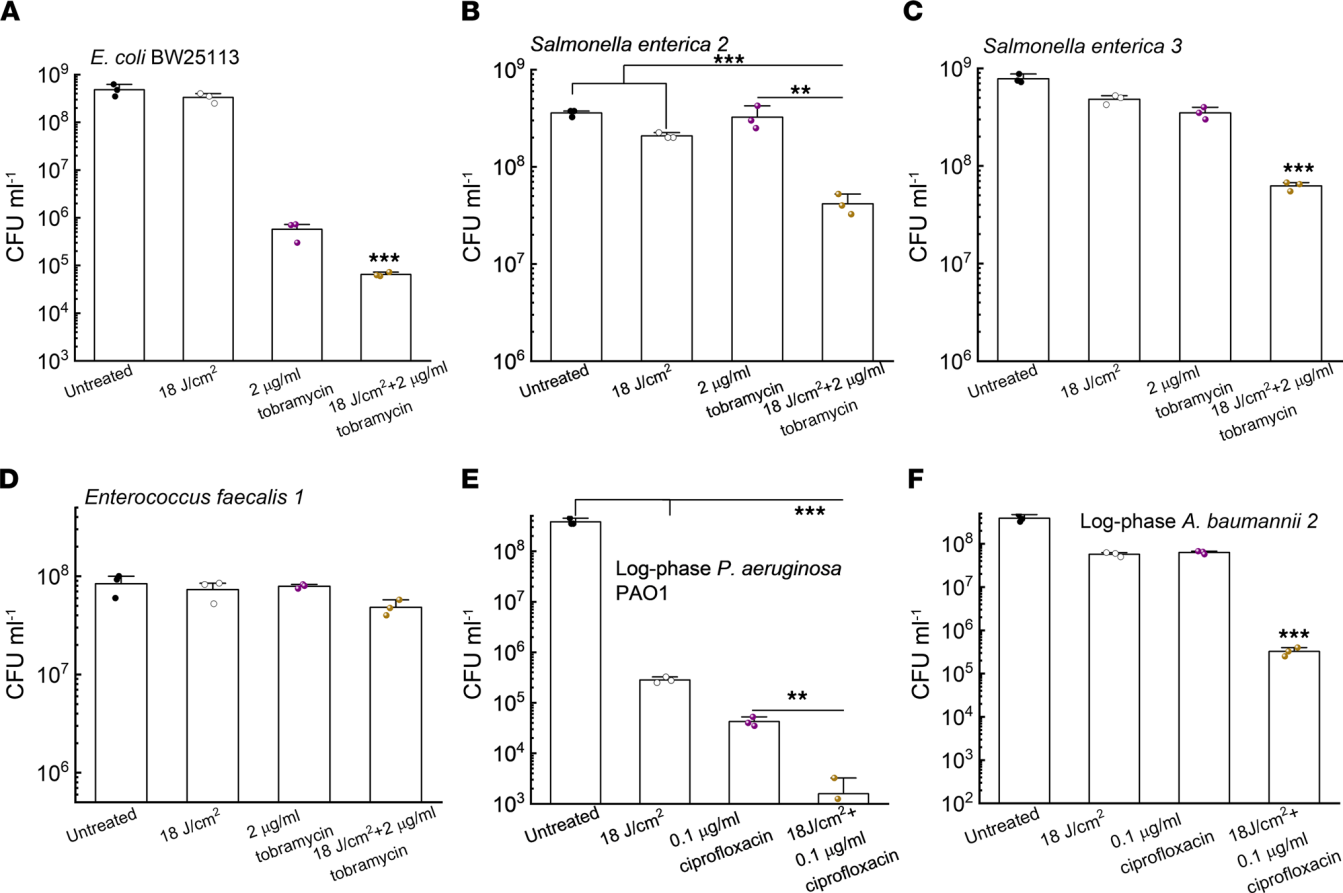
Photoinactivation of catalase sensitizes pathogenic bacteria to certain antibiotics. (**A**) CFU/mL of *E*. *coli* BW25113 under different treatments. (**B** and **C**) CFU/mL of *S*. *enterica* under different treatments. CFU enumeration was obtained by incubation with tobramycin in TSB for 4 hours. (**D**) CFU/mL of *E*. *faecalis 1* under the same treatments as in **B** and **C**. (**E** and **F**) CFU/mL of log-phase *P*. *aeruginosa* PAO1 (**E**) along with *A*. *baumannii* *2* (**F**) under different treatment schemes. CFU enumeration was achieved after incubation with ciprofloxacin in TSB for 4 hours. Data: Mean + SD from 3 replicates for all the panels. Statistical analysis was determined by Student’s 2-tailed unpaired *t* test and 1-way ANOVA. ***P < 0.001, **P < 0.01. Significant difference from other groups unless notified.

Significant intracellular H_2_O_2_ is produced upon treatment with ampicillin (β-lactam), gentamicin (aminoglycoside), and norfloxacin (fluoroquinolone; ref. 38). Based on these prior studies, we reasoned that photoinactivation of catalase could enhance the efficacy of these antibiotics that induce ROS generation. To test our hypothesis, we first tested tobramycin, a representative of aminoglycoside that could indirectly enhance the intracellular production of H_2_O_2_ (48). Augmented killing effect was observed with 410 nm exposure plus tobramycin treatment of *E*. *coli* BW25113 compared with tobramycin treatment alone (Figure 4A). The 410 nm exposure strengthened the bactericidal effect of tobramycin against *S. enterica* by around 1-log_10_ (Figure 4, B and C) and did not enhance tobramycin killing of catalase-negative *E. faecalis 1* (Figure 4D).

Per report, catalase-deficient *E*. *coli* mutants are more susceptible to fluoroquinolone ciprofloxacin when compared with their parent strain (49). We thus wondered whether photoinactivation of catalase could sensitize bacteria to ciprofloxacin. As shown in Figure 4E, photoinactivation of catalase indeed boosted the antimicrobial potency of ciprofloxacin to eliminate log-phase *P*. *aeruginosa* PAO1. Similar enhancement was observed for log-phase *A*. *baumannii 2* (Figure 4F). Collectively, these findings suggest that photoinactivation of catalase augments the antimicrobial efficacy of at least some antibiotics that indirectly increase the intracellular H_2_O_2_ level.

### Photoinactivation of catalase and H_2_O_2_ synergistically eliminate P. aeruginosa biofilms.

Having shown effective synergy between photoinactivation of catalase and ROS-generating agents against both log- and stationary-phase planktonic bacteria, we next asked if the synergy can effectively eradicate biofilm-dwelling bacteria.

To address our query, we selected *P*. *aeruginosa* as a target since it is a notorious pathogen in burn wounds, chronic obstructive pulmonary disorder, and cystic fibrosis (50). Extensive *P*. *aeruginosa* biofilm protects the bacterium from host defense, chemotherapy, and conventional antimicrobial therapy, leading to undesirable disease burden on patients (51, 52).

**Figure 5:**
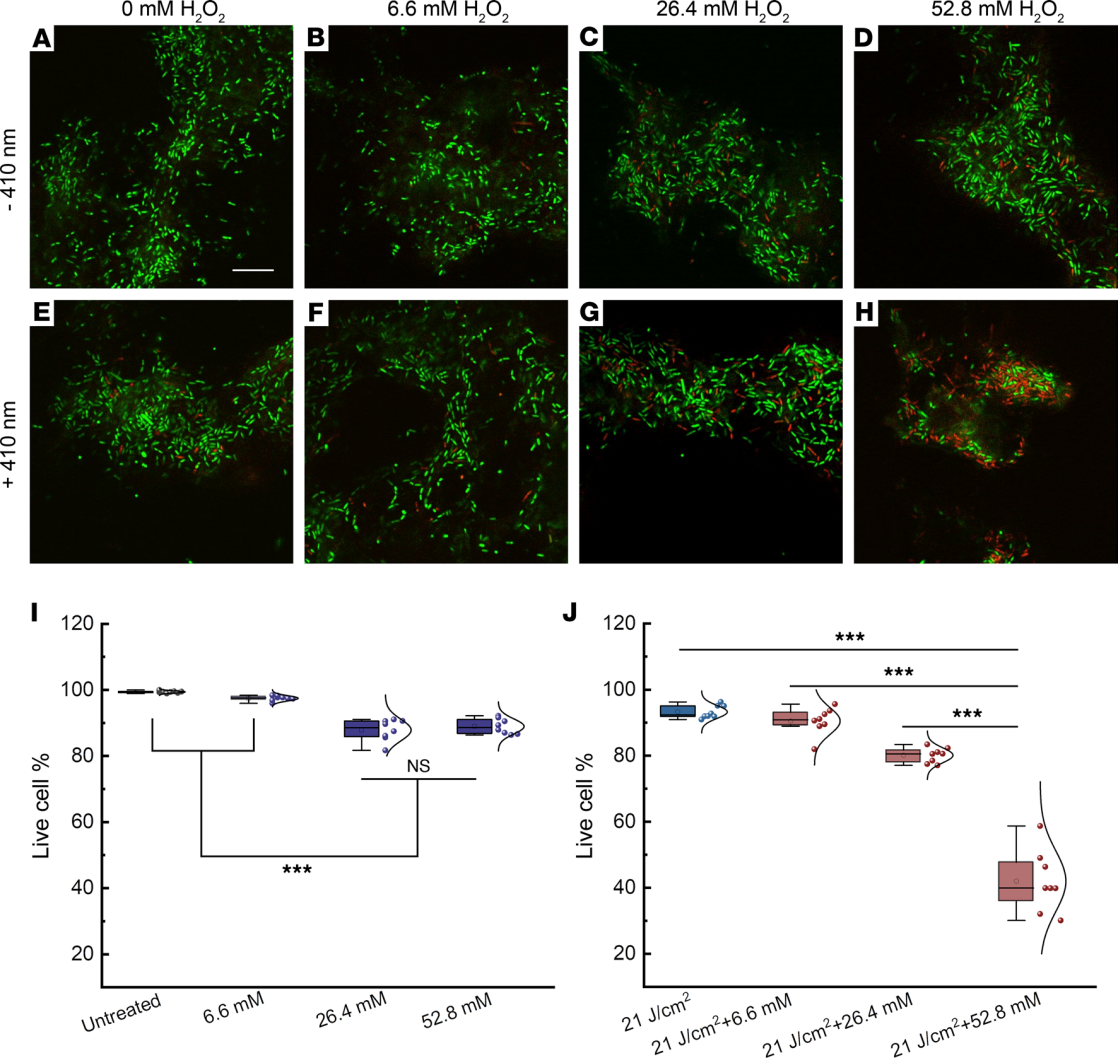
Confocal laser scanning microscopy of live/dead bacteria inside *P*. *aeruginosa* PAO1 biofilms after different treatments. (**A**–**D**) Merged live/dead *P*. *aeruginosa* biofilms with various H_2_O_2_ treatments. (**E**–**H**) Merged live/dead *P*. *aeruginosa* biofilms after 410 nm exposure combined with various H_2_O_2_ treatments. (**I** and **J**) Quantitative analysis of the live-cell percentage of *P*. *aeruginosa* biofilms among the above 8 groups. Live-cell percentage was calculated from 8 different fields of view. Box-and-whisker plots: mean, circle; median, horizontal line; box range, percentiles 25, 75. Scale bar: 10 μm. Live: SYTO 9. Dead: PI. 410 nm laser: ns-410 nm, 35 mW, 10 minutes. H_2_O_2_: 30-minute incubation time. Statistical analysis was determined through Student’s 2-tailed unpaired *t* test and 1-way ANOVA. ***P < 0.001.

To mimic clinical *P*. *aeruginosa* infections in which biofilm has a major presence, we adopted a CDC biofilm growth protocol that grows *P*. *aeruginosa* biofilms on a polypropylene coupon under continuous-flow conditions (53). After forming robust biofilms, we applied treatments (untreated, H_2_O_2_ at a series of concentrations, 410 nm exposure [21 J/cm^2^], 410 nm [21 J/cm^2^] plus H_2_O_2_ [30-minute incubation time]). Then we used a Live (SYTO 9)/Dead (propidium iodide, PI) staining kit to image live/dead *P*. *aeruginosa* through a confocal microscope. As shown in Figure 5, A–D, H2O_2_-treated groups barely had a noticeable number of dead cells compared to the untreated group (Figure 5I). In contrast, a drastic increase of dead bacteria number was observed in groups treated with 410 nm exposure plus H_2_O_2_ (Figure 5, E–H and J). Three-dimensional rendered images of *P*. *aeruginosa* biofilms (Supplemental Video 2) further corroborated the synergistic effect between 410 nm exposure and H_2_O_2_ treatment to eliminate *P*. *aeruginosa* biofilms.

Of note, even after combining 410 nm and H_2_O_2_ at a concentration of 52.8 mM, full eradication still could not be achieved (around 70% elimination, Figure 5, E–H and J), which may be attributed to the shielding conferred by the extracellular polymeric substances or quorum sensing inside the biofilms. Nonetheless, photoinactivation of catalase and H_2_O_2_ substantially eliminated *P*. *aeruginosa* in the biofilm setting, which suggests the potential for treating clinically relevant *P*. *aeruginosa* infections, such as burn infections.

### Photoinactivation of catalase assists macrophages to eliminate intracellular pathogens.

Besides forming persistent biofilms, bacteria could also reside inside host immune or nonimmune cells to evade antibiotic attack (54, 55). It has been reported that the minimal inhibitory concentrations of intracellular MRSA are 2 orders of magnitude higher than those of free-living planktonic bacteria (55). Furthermore, *S*. *aureus* can proliferate within host phagocytic cells, such as neutrophils and macrophages, shortly after intravenous infection (56, 57). These viable intracellular *S*. *aureus* allow the infected cells to act as Trojan horses for further dissemination to cause systematic infections (58). Therefore, more effective elimination of intracellular bacteria has the potential to improve current antibiotic therapeutics.

Staphylococcal catalase shields intracellular *S*. *aureus* by degrading H_2_O_2_ generated by murine peritoneal macrophages (59). Catalase has been reported to help *Campylobacter jejuni* survive within macrophages as evidenced by extensive killing of catalase-deficient *Campylobacter jejuni* by macrophages (60). Neutrophils, as the first line of defense, can also harbor pathogens inside the phagocytic vacuoles (61), abetted by bacterial catalase that plays a vital role in intracellular survival (62). Therefore, we wondered whether photoinactivation of catalase could boost immune mechanisms of eliminating intracellular pathogens.

**Figure 6:**
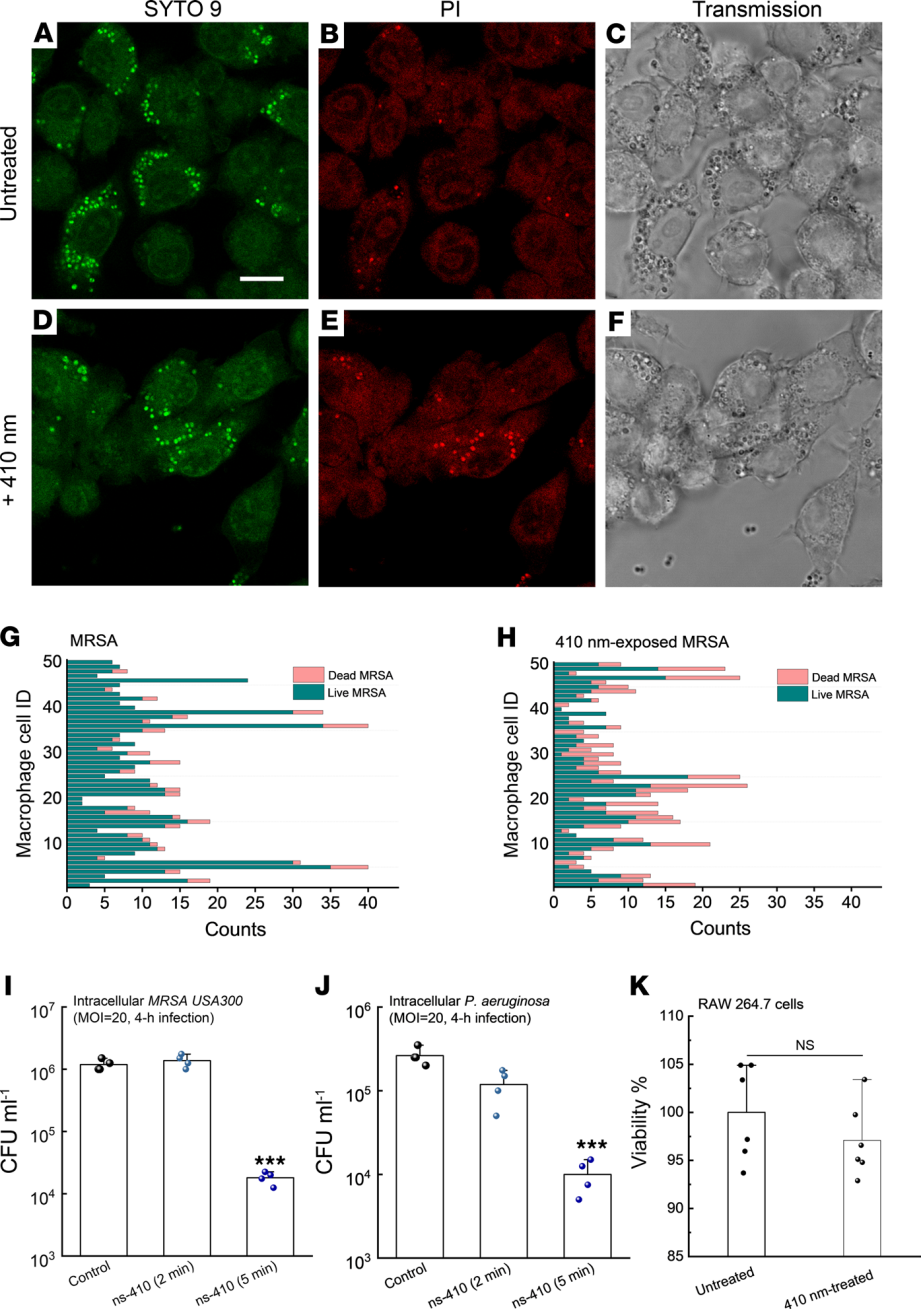
Photoinactivation of catalase assists macrophages to eliminate intracellular bacteria. (**A**–**C**) Confocal images of live (SYTO 9, **A**), dead (PI, **B**), and corresponding transmission images (**C**) of intracellular MRSA USA300 inside RAW 264.7 macrophages after MRSA USA300 infected RAW 264.7 cells for 1 hour at a MOI of 100 in serum-free DMEM. (**D**–**F**) Confocal images of live (SYTO 9, **D**), dead (PI, **E**), and corresponding transmission images (**F**) of intracellular MRSA USA300 inside RAW 264.7 macrophages after 410 nm–exposed MRSA USA300 infected RAW 264.7 cells for 1 hour at an MOI of 100. 410 nm: 35 mW/cm^2^, 8 minutes. (**G** and **H**) Quantitative analysis of the amount of live/dead MRSA inside single RAW 264.7 cells from the above 2 scenarios. Imaging result was a representative of 2 biological repeats. (**I**) CFU/mL of intracellular MRSA USA300 after MRSA (with/without 410 nm exposure) infected RAW 264.7 cells for 4 hours at an MOI of 20. (**J**) CFU/mL of intracellular *P*. *aeruginosa* after *P*. *aeruginosa* PAO1 (with/without 410 nm exposure) infected RAW 264.7 cells for 4 hours at an MOI of 20. (**K**) Survival percentage of uninfected RAW 264.7 cells with/without 410 nm exposure. 410 nm: 50 mW/cm^2^. Data: Mean + SD from 3 replicates for panels **I**–**K**. Significant difference was determined by 1-way ANOVA (significant from other 2 groups). ***P < 0.001.

Thus, we infected the mouse macrophage cell line RAW 264.7 with log-phased MRSA USA300 and 410 nm pretreated log-phase MRSA USA300 at a multiplicity of infection (MOI) of 100 for 1 hour. After eliminating MRSA USA300 outside the macrophages by incubating with gentamicin for 1 hour, we adopted a protocol to label the live (SYTO 9)/dead (PI) MRSA USA300 inside the macrophages (63). As shown in Figure 6, A–C, there was a large number of live MRSA USA300 in the cytoplasm of macrophages after phagocytosis of untreated MRSA USA300. In comparison, the number of dead 410 nm preexposed MRSA USA300 was markedly increased inside macrophages (Figure 6, D–F). We performed quantitative analysis of live/dead MRSA USA300 inside single macrophages from 50 macrophages (Figure 6, G–H). There was a clear difference in the number of dead bacteria between the 2 groups.

To confirm that photoinactivation of catalase enhances macrophage elimination of intracellular pathogens, we enumerated intracellular bacteria by lysing macrophages with 0.1% Triton X-100 for 3 minutes after 4-hour infection. As shown in Figure 6I, there was an approximate 2-log_10_ reduction of intracellular MRSA USA300 burden in the 410 nm exposure (15 J/cm^2^) group. Consistently, a 1.5-log_10_ reduction of intracellular *P*. *aeruginosa* was obtained in macrophages infected with 410 nm (15 J/cm^2^) exposed *P*. *aeruginosa* (Figure 6J). In addition, we tried to treat macrophages with blue light after internalization of *P*. *aeruginosa*; the 410 nm–treated group consistently demonstrated lower intracellular bacterial burden (Supplemental Figure 5A). Also, we found that use of an NADPH oxidase (NOX) inhibitor diminished this difference (Supplemental Figure 5B), which suggests that ROS burst inside macrophages is crucial for efficient elimination of intracellular bacteria. We showed that 410 nm exposure did not cause significant toxicity to noninfected RAW 264.7 cells (Figure 6K). Collectively, the results of the confocal live/dead imaging along with the CFU assay suggest that 410 nm exposure can facilitate host immune killing of intracellular pathogens.

### Photoinactivation of catalase reduces P. aeruginosa burden in a mouse skin abrasion model.

**Figure 7:**
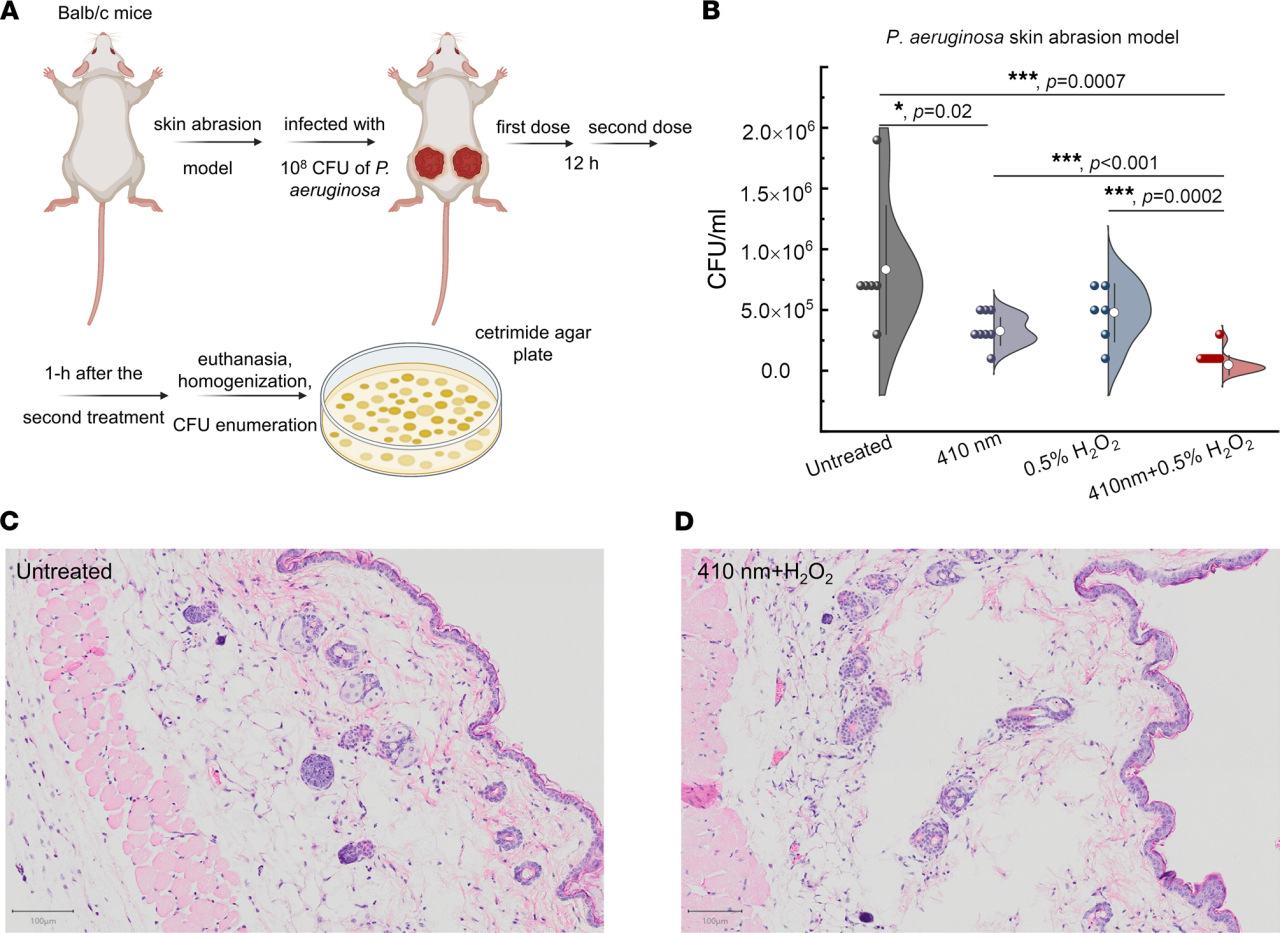
Photoinactivation of catalase reduces *P*. *aeruginosa* burden in a *P*. *aeruginosa*–induced mouse skin abrasion model. (**A**) Schematic illustration of in vivo mouse experiment. (**B**) CFU/mL of *P*. *aeruginosa* PAO1 from the infected wound tissues among 4 different groups. (**C** and **D**) Histology analysis of mouse skin from untreated group along with 410 nm plus H_2_O_2_ treated group. Scale bar: 100 μm. Data: Mean ± SD from at least 6 replicates (*n* = 3–4 mice/group, 6–8 infected area/group). 410 nm: 120 J/cm^2^. H_2_O_2_: 0.5%. Significant difference was determined by Student’s 2-tailed unpaired *t* test and 1-way ANOVA. ***P < 0.001, *P < 0.05. An outlier was removed based on Dixon’s Q test and the box-and-whisker plot.

We further evaluated the synergy between photoinactivation of catalase and H_2_O_2_ using a bacterial infection murine model. Specifically, we adopted a *P*. *aeruginosa*–infected mouse skin abrasion model (64) to mimic clinical *P*. *aeruginosa*–mediated skin infection. Briefly, we applied 10^8^ CFU of *P*. *aeruginosa* to abraded mouse skin for 3 hours. After the wound was established, 410 nm blue light (120 J/cm^2^), 0.5% H_2_O_2_, and 0.5% H_2_O_2_ plus 410 nm (120 J/cm^2^) were independently applied to the infected area 2 times before euthanasia (Figure 7A). Wound tissues from the euthanized mice were then subjected to homogenization in order to enumerate CFU. As shown in Figure 7B, the untreated mice’s wounds had around 10^6^ CFU/mL of *P*. *aeruginosa*, and 410 nm light alone significantly (*P* = 0.02) reduced bacterial burden by around 60%. Of note, 410 nm light exposure significantly enhanced (*P* = 0.0002) the bactericidal effect of 0.5% H_2_O_2_ against *P*. *aeruginosa* by 1 order of magnitude.

To evaluate whether our combinational treatment causes skin damage, we collected the mouse skin after applying 410 nm at the same dose plus 0.5% H_2_O_2_. We then performed the hematoxylin and eosin (H&E) staining and histology analysis. As shown in Figure 7, C and D, epidermis, dermis, and subcutaneous tissues were not different from the untreated control.

To evaluate whether longitudinal blue light treatment could generate any effect on mouse physiology, we applied 3-consecutive-day blue light treatment after *P*. *aeruginosa* infection (once a day). Meanwhile, wound size, body temperature, and weight were monitored and recorded each day after treatment. As shown in Supplemental Figure 6, we did not observe significant changes in the mice’s temperature and body weight among different treatment groups over time. Interestingly, there was a significant difference regarding the wound size (Supplemental Figure 6C) between the untreated group and combination (410 nm plus H_2_O_2_) treated group. The combination-treated group showed smaller wound size and better wound-healing outcome. Our data further indicated that mice did not develop bacteremia 3 days after the infection, even in the untreated group, as their activity and mobility appeared normal.

Collectively, these in vivo data provide the foundation for exploring the clinical utility of our catalase-targeted therapy against multidrug-resistant bacterial infections.

## Discussion

Since the discovery of penicillin in 1928 by Alexander Fleming (65), there was a golden age (from the 1940s to 1960s) for the discovery of antibiotics, most of which remain in clinical use today (66). Yet, imprudent usage of antibiotics sped up the natural selection of drug-resistant bacteria (67). In the past decades, the ever-rising emergence of multidrug-resistant bacteria has been an alarming threat worldwide. Moreover, the pace of antibiotic development has not kept up with antibiotic resistance development. Therefore, novel alternative approaches are highly desired to combat the new waves of multidrug-resistant bacterial infections.

It has been reported that catalase serves an important virulence function for intracellular bacteria evasion of neutrophil phagocytosis (68). *E*. *coli* pretreated with phenazine methosulfate showed a 9-fold increase of catalase synthesis and demonstrated resistance against neutrophil killing (69). With catalase expression, *S*. *aureus* has significantly higher viability in the presence of human neutrophils (70). Besides neutrophils, staphylococcal catalase protects intracellular bacteria by neutralizing H_2_O_2_ produced by macrophages (59). Not only does catalase plays an important role in host-microbe interaction, but it has also been shown that catalase expression contributes significantly to the survival of catalase-positive *S*. *aureus* against catalase-negative *Streptococcus pneumoniae* in a murine model of nasal colonization (71). Therefore, catalase inactivation could deprive pathogenic bacteria of an essential armament.

In this paper, we identified catalase as a key molecular target of blue light for a wide range of pathogens. Through spectroscopic study, we found that catalase can be functionally inactivated by blue light, especially at 410 nm. We further demonstrated that photoinactivation of catalase renders wide-ranging pathogenic bacteria and *P*. *aeruginosa* biofilms highly susceptible to subsequent H_2_O_2_ or H_2_O_2_-producing agents. The close correlation between catalase inactivation and its bacterial killing efficiency, the catalase gene complementation study, further validate that catalase is a major molecular target of blue light. In addition, photoinactivation of catalase significantly enhances macrophage killing of intracellular pathogens and further reduces bacterial burden in a *P*. *aeruginosa*–infected mouse abrasion model without causing significant skin damage. Toward clinical translation, the synergy between photoinactivation of catalase and H_2_O_2_/antibiotics is most suitable for superficial bacterial infections due to limited penetration depth of 410 nm light. To improve the treatment depth, tissue-clearing agents can be applied to enhance the light penetration through the skin. Alternatively, up-conversion nanoparticles can be employed by tuning the excitation wavelength from blue light to near infrared light (72). For intrabody infections such as urinary tract infections, light-emitting catheters can be designed to deliver the blue light to the infection site.

We found that ns-410 nm blue light was substantially more effective at inactivating catalase than CW-410 nm using the same dose. Moreover, ns-410 nm plus H_2_O_2_ killed more bacteria compared with CW-410 nm plus H_2_O_2_. Enhanced bacterial killing probably came from the second-order photobleaching behavior of catalase under 410 nm irradiance, where prosthetic heme rings inside the tetramer might react with each other to detach from protein matrix. Raman spectra of catalase before and after 410 nm treatment hinted at porphyrin changes or dissociation. As an additional benefit, ns-410 nm exposure could also eliminate the heating issue that is always accompanied by CW-410 nm irradiance. It has been reported that heat dissipation is in the range of microseconds (73), and ns-410 nm here was modulated at a frequency of 10 Hz, which could efficiently address the heat accumulation issue. In short, ns-410 nm might have better potential for clinical translation to treat multidrug-resistant bacterial infections compared with CW-410 nm.

We also found that stationary-phase pathogens such MRSA USA300 or *P*. *aeruginosa* were markedly more susceptible to subsequent H_2_O_2_ attack after photoinactivation of catalase. Stationary-phase and nondividing pathogens (e.g., persisters) are known to cause persistent infections such as endocarditis or osteomyelitis or biofilm-associated infections (74). The persisting pathogens appear to be metabolically dormant and thrive under nutrition depleted environment (75). The dormant bacteria demonstrate higher levels of tolerance and persistence to antimicrobial agents when compared to metabolically active planktonic bacteria (76). Therefore, alternative approaches to combat stationary-phase pathogens and persisters are important. Catalase activity has been reported to be increased in some stationary-phase bacteria (77). Our potentially novel approach demonstrated bactericidal efficacy against a broad range of stationary-phase pathogens. Thus, we believe this synergistic treatment will presumably work on the persisters as well.

We showed that photoinactivation of catalase also enhances immune cell elimination of intracellular pathogens. Bacterial catalase promotes intracellular pathogens’ survival inside immune cells (78). As shown in Figure 6A, a substantial portion of MRSA USA300 remained alive after internalization by macrophages. After photoinactivation of catalase, both intracellular MRSA USA300 and *P*. *aeruginosa* burden were significantly reduced. Photoinactivation of catalase also significantly reduced bacterial load in the mouse skin abrasion model (Figure 7B) without causing skin damage. Although catalase is a major molecular target of blue light, it is not the only target of 410 nm irradiance since some H_2_O_2_ killing of catalase-deficient *E*. *coli* mutants and *S*. *aureus* mutants was still observed after 410 nm blue light treatment (Figure 2). This finding indicates that molecular targets of the 410 nm light other than catalase likely exist. Of note, there are other endogenous molecules that are intrinsic to specific bacteria already revealed as molecular targets of blue light. For example, researchers have demonstrated endogenous pigments are essential virulence factors for microbes (79). Moreover, many of them can be inactivated by photons, thus rendering microbes highly susceptible to exogenous ROS (16, 17). Therefore, intrinsic pigments can be explored to find out other molecular targets. Another intriguing direction is to interrogate the possible correlation between catalase level and drug resistance. If drug-resistant bacteria show the propensity to express a higher level of catalase compared with drug-susceptible bacteria, photoinactivation of catalase might provide a universal approach to disarm drug-resistant pathogens. Collectively, our findings reveal a mechanism of antimicrobial blue light and fuel a catalase-targeted strategy to combat clinical multidrug-resistant bacterial infections.

## Methods

### Blue light source.

Pulsed blue light was administered using an Opolette HE 355 LD laser (OPOTEK). The nanosecond-pulsed laser is tunable from 410 nm to 2200 nm, has a repetition rate of 10 to 20 Hz, and has a pulse width of approximately 5 to 10 ns. Using a collimator attached to an optical fiber, the diameter of the laser beam was expanded to 10 mm. With these set parameters, the pulsed laser provides a power output ranging from 25 to 100 mW/cm^2^, depending on the laser wavelength used. CW blue light was delivered through a mounted 405 nm blue light LED (M405L4, Thorlabs) with an adjustable collimation adapter (SM2F32-A, Thorlabs) focusing the illumination region to an approximately 1 cm^2^ region. A T-Cube LED driver (LEDD1B, Thorlabs) allowed for adjustable light fluencies up to 400 mW/cm^2^.

### Bacterial strains and cell lines.

Bacterial strains MRSA (USA300), *P*. *aeruginosa* PAO1 (ATCC 47085), *P*. *aeruginosa* 2 (ATCC 1133), *P*. *aeruginosa* 3 (ATCC 15442), *P*. *aeruginosa* 4 (ATCC 9027), *S*. *enterica* 2 (ATCC 700720), *S*. *enterica* 3 (ATCC 13076), *E*. *coli* 1 (BW25113), *Klebsiella* *pneumoniae* (*K*. *pneumoniae* 1, ATCC BAA 1706), *A*. *baumannii* 1 (ATCC BAA 1605), *A*. *baumannii* 2 (ATCC BAA-747), *E*. *faecalis* 1 (NR-31970), and *E*. *faecalis* 2 (HM-335) were provided by Mohamed N. Seleem at Virginia Tech University, Blacksburg, Virginia, USA. *E*. *coli* mutants (*ΔahpC*, *ΔkatG*, *ΔkatE*, *ΔkatGE*) were provided by the Xilin Zhao group at Rutgers University, New Brunswick, New Jersey, USA. All cell lines used in this study, including the RAW 264.7 murine macrophages and CHO cells, were purchased directly from the ATCC.

### Transient absorption imaging of real-time photobleaching of catalase.

As described previously (80), an optical parametric oscillator synchronously pumped by a femtosecond pulsed laser generated the pump (820 nm) and probe (1040 nm) pulse trains. The pump and probe beams were then frequency-doubled via the second–harmonic generation process to 410 nm and 520 nm through barium borate crystals, respectively. Temporal delay between the pump and probe pulses was controlled through a motorized delay stage. The pump beam intensity was modulated with an acousto-optic modulator. The intensity of each beam was adjustable through the combination of a half-wave plate and a polarization beam splitter. Thereafter, pump and probe beams were collinearly combined and directed into a laboratory-built laser scanning microscope. Through the nonlinear process in the sample, the modulation of the pump beam was transferred to the unmodulated probe beam. Computer-controlled scanning galvo mirrors were used to scan the combined laser beams in a raster scanning approach to create microscopic images. The transmitted light was collected by an oil condenser. Subsequently, the pump beam was spectrally filtered by an optical filter, and the transmitted probe intensity was detected by a photodiode. A phase-sensitive lock-in amplifier (Zurich Instruments) then demodulated the detected signal. Therefore, pump-induced transmission changes in the probe beam versus the temporal delay can be measured. This change over time delay shows different time-domain signatures of a chromophore, thus offering the origin of the chemical contrast.

### Raman spectra of catalase before and after 410 nm irradiance.

Raman spectra of catalase before and after 410 nm exposure were acquired under a Horiba Raman system (1221, LABRAM HR EVO) with a 40× objective under the excitation of 532 nm. Raman spectra were obtained under a 20-second acquisition time with a 10% laser ND filter. Bovine liver catalase (C9322, MilliporeSigma) was dissolved in sterile distilled H_2_O (4 U/mL) and then air-dried onto a clean cover slide. Changes in catalase structure were evaluated by examining decreases in specific Raman peaks following light treatment.

### Quantitation of remaining active catalase.

Measurement of active catalase was primarily quantified through the use of an Amplex Red Catalase Assay (A22180, Thermo Fisher Scientific). Briefly, solutions containing catalase (either bovine liver catalase or catalase-positive bacteria inoculum) were treated with blue light, after which 25 μL of the light-treated solution was incubated with 40 μM of H_2_O_2_ for 30 minutes at room temperature within a 96-well plate. Following H_2_O_2_ treatment, 50 μL of a reaction stock containing 100 μM of Amplex Red and 0.4 U/mL of horseradish peroxidase were added to each well, and the combination was incubated for 30 minutes at 37°C. Following incubation, the fluorescence of each well was measured using an excitation wavelength of 543 nm and an emission wavelength of 585 nm. In addition to the light-treated samples, a PBS negative control and an untreated positive control were treated with the assay to determine the remaining catalase percentage. Active catalase percentage was calculated through the following equation: remaining catalase% = ([I_PBS_ – I_treated_]/[I_PBS_ – I_untreated_]) × 100%.

### CFU enumeration assay to evaluate the synergy between photoinactivation of catalase and ROS-generating agents.

*P*. *aeruginosa* and MRSA were cultured overnight in Tryptic Soy Broth (TSB) at 37°C within a shaking incubator (250 rpm). The next day, the bacteria were suspended in 1× PBS (OD_600_ = 1.0), after which a 10 μL aliquot was placed on a glass cover slide and then treated with ns-410 nm or CW-410 nm. Following light treatment, the droplet was transferred to a tube containing either PBS or 22 mM of H_2_O_2_ diluted in PBS. Samples were incubated for 30 minutes under 37°C, after which the bacteria samples were 10-fold serially diluted within a 96-well plate, plated on tryptic soy agar plates overnight, and then CFU enumerated the next day. Subsequent experiments testing the synergy between ns-410 or CW-410 blue light and different concentrations of H_2_O_2_ were also performed on other stationary-phase bacteria, including *E*. *coli*, *P*. *aeruginosa*, MRSA, *A*. *baumannii*, *S*. *enterica*, *K*. *pneumoniae*, and *E*. *faecalis*. Similar to previous CFU synergy experiments, wild-type *E*. *coli* (BW25113), an alkyl hydroperoxide reductase-negative mutant strain (*ΔahpC*), and 3 catalase-negative mutants (*ΔkatG*, *ΔkatE*, *ΔkatGE*) were all cultured overnight in TSB at 37°C within a shaking incubator (250 rpm). Following incubation, bacteria strains were suspended in 1× PBS (OD_600_ = 1.0). A 20 μL aliquot of bacteria was placed on a glass coverslip and exposed to ns-410 light (32 mW/cm^2^, 14 J/cm^2^). The droplet was then removed and diluted with 780 μL of PBS. This light-treated sample, alongside a non-light-exposed sample, was treated with 2.2 mM of H_2_O_2_. Samples were shaken and incubated at 37°C for up to 2 hours. During the incubation, 60 μL aliquots would be removed from each sample tube for serial dilatation and CFU plating at the 20-minute, 40-minute, 1-hour, 1.5-hour, and 2-hour time points.

### Mammalian cell toxicity assay.

To evaluate the potential toxicity of 410 nm exposure and short-term, high-concentration H_2_O_2_ exposure against mammalian cells, an MTT assay was performed using CHO cell line (ATCC) and noninfected RAW 264.7 cells. RAW 264.7/CHO cells were cultured in DMEM (Gibco) alongside 10% FBS until 90% confluence was achieved. Once high confluence was established, cells were removed through trypsin treatment, quantified with a cell counter, and diluted in DMEM to a final cell concentration of 1 × 10^6^ cells/mL. A total of 100 μL of cell media were added to each well of a 96-well plate, providing each well with 1 × 10^5^ cells. Each well was supplemented with an additional 100 μL of DMEM to bring the final volume of each well to 200 μL. HEK/CHO cells were then incubated overnight at 37°C with 5% CO_2_ in order to allow the cells to adhere to the wells. Following the replacement of DMEM with PBS, light-treated wells were exposed to 75 J/cm^2^ of 410 nm LED light (250 mW/cm^2^). After light exposure, PBS was removed and replaced with DMEM. For the H_2_O_2_ treatment groups, 0.1% H_2_O_2_ suspended in DMEM was added to the H_2_O_2_ treatment groups for 1 minute, after which the H_2_O_2_-containing media was removed and the wells were washed twice with PBS. Identical 2-fold PBS washing was also applied to the other treatment groups to maintain experimental consistency. After that, the PBS was replaced with fresh DMEM and incubated overnight at 37°C with 5% CO_2_ to allow for the surviving cells to recover from the stress exerted by the treatment. The next day, an MTT viability assay was performed based on previously established protocols. MTT absorbance measurements were quantified via plate reader at 590 nm. The assay was performed in replicates of *n* = 4.

### Preparation of catalase rescue strain E. coli ΔkatGE:pBad_katE.

An *E*. *coli* DH5α strain expressing an arabinose-regulated pBAD-HPII (katE catalase) plasmid (Addgene, 105839) was cultured, and a GeneJET Plasmid Miniprep Kit (Thermo Fisher Scientific, K0503) was used to isolate the pBAD-HPII plasmid. A TransformAid Bacterial Transformation Kit (Thermo Fisher Scientific, K2711) was then used to transform the double catalase knockout of *E*. *coli* *ΔkatGE* and insert the arabinose-regulated catalase plasmid to form a catalase rescue strain *E*. *coli* *ΔkatGE*:*pBad_katE*. Then both wild-type *E*. *coli* BW25113 and *E*. *coli* *ΔkatGE*:*pBad_katE* were cultured at 37°C to log phase and then resuspended to PBS. A 10 μL aliquot was treated to CW-410 (30 J/cm^2^) and then incubated in a M9 minimal media containing 1% arabinose for 4 hours at 37°C. Following the incubation, the bacterial suspension was treated with 2.2 mM of H_2_O_2_ for 30 minutes and then plated on agar plates. CFU enumeration was recorded for phenotypic response comparisons.

### CFU assay between photoinactivation of catalase and certain antibiotics.

For each antibiotic tested, bacterial strains were usually precultured in antibiotics prior to light exposure of subsequent incubation. To summarize, 1 mL of overnight-cultured bacteria was centrifuged and suspended in 1 mL of fresh TSB media supplemented with either 10 μg/mL of tobramycin (MilliporeSigma, T4014) or 0.1 μg/mL of ciprofloxacin (MilliporeSigma, 17850). Following preculture treatment, the bacterial solution was spun down and resuspended in 1× PBS. A 10 μL aliquot of the bacterial preculture stock was then placed on a coverslip and exposed to ns-410 with a dose of 18 J/cm^2^, after which the aliquot was transferred to 990 μL of fresh TSB supplemented with either 2 μg/mL of tobramycin or 0.1 μg/mL of ciprofloxacin and incubated at 37°C for up to 6 hours. CFU dilution and enumeration was taken at various time points during the incubation period.

### Intracellular bacteria assay.

In all experiments, bacteria were cultured in TSB. To assess intracellular killing with the macrophage RAW 264.7 cell line, MRSA USA300 or *P*. *aeruginosa* was taken from an exponentially growing culture and washed in PBS. Macrophages were prewashed with serum-free DMEM immediately before infection and infected by MRSA USA300/*P. aeruginosa* with and without 410 nm treatment. Then coculture was incubated at 37°C in a humidified tissue culture incubator with 5% CO_2_ to permit phagocytosis of the bacteria. After 2 to 4 hours, the infection mix was removed and replaced with normal growth media (DMEM supplemented with 10% FBS, 10 mM HEPES), and gentamicin was added at 50 μg/mL to prevent growth of extracellular bacteria for 1 hour. Two approaches were used to evaluate the difference. The first one was to utilize a Live/Dead confocal staining assay to visualize the live and dead bacteria inside macrophages, respectively. Briefly, after fixation with 10% formalin following gentamicin treatment, samples were permeabilized with 0.1% Triton X-100 (MilliporeSigma) for 3 minutes at room temperature. After that, a Live/Dead fluorescence kit (Thermo Fisher Scientific, L7007) was used to stain the intracellular bacteria. A confocal laser scanning microscope (FV3000, Olympus) was used to visualize stained samples. The second one was to enumerate the intracellular CFU. In brief, cocultures were permeabilized with 0.1% Triton X-100 for 3 to 5 minutes, and vigorous pipetting was conducted to release intracellular bacteria. Ten-fold serial dilution was immediately applied to count the viable intracellular bacteria.

To quantify the impact of NOX inhibitor on elimination of intracellular bacteria by macrophages, macrophages were first exposed to diphenyleneiodonium chloride (DPI, MilliporeSigma, D2926) for 1 hour. DPI is a known inhibitor of the NOX enzymes responsible for the ROS burst in macrophages. After that, *P*. *aeruginosa* with/without 410 nm exposure infected macrophages at an MOI of 20. Two hours after infection, gentamicin was added to the coculture to remove extracellular bacteria for 1 hour. Then intracellular bacteria were quantified after permeabilization with 0.1% Triton X-100 for 3 minutes and serial dilution.

### P. aeruginosa biofilm assay.

As reported previously (10), *P*. *aeruginosa* biofilms were formed onto a coupon through a CDC biofilm reactor for 2 days. After the biofilm formation, different treatment schemes were applied. Live/dead fluorescence staining was used to evaluate the treatment efficacy. A confocal laser scanning microscope was then used to capture the counterstained images.

### In vivo murine infection model and histology.

Twenty BALB/c mice (Jackson Laboratories, 000651) were placed under anesthesia, and a #15 sterile scalpel was used to generate a 1 cm^2^ abrasion wound by carefully scraping the epidermis of the skin without drawing blood. Once the wound had been generated, a 10 μL aliquot containing 10^8^ CFU of log-phase PAO1 in PBS was placed onto the abrasion wound and spread evenly across the wound with a pipette tip. Once the droplet had dried, the mice were returned to their cage to allow them to recover from their abrasion wound and to provide time for the bacteria to infect the wound. The 20 mice were then divided into 4 treatment groups, each consisting of 5 mice (*n* = 5): untreated, 410 nm treated, H_2_O_2_ treated, and 410 nm plus H_2_O_2_ treated. Light treatment was applied by positioning mice under 200 mW/cm^2^ (ANSI standard) 410 nm LEDs and exposing their bacteria-infected wounds to 120 J/cm^2^ of blue light. For H_2_O_2_ treatment, 10 μL of 0.5% H_2_O_2_ was evenly distributed on the infected wounds and allowed to naturally dry. Combination treatment consisted of the application of previously described light treatment followed by H_2_O_2_ treatment. Treatments were applied to mice twice over the course of 15 hours, with the first treatment being applied 3 hours following infection and the second treatment being applied 12 hours after the first treatment. 3 hours after the second treatment, mice were euthanized and wound tissue was harvested, homogenized, and serially diluted. CFU enumeration was performed on *P*. *aeruginosa*–specific cetrimide agar plates (MilliporeSigma, 22470).

The potential phototoxicity of the treatment on the skin was evaluated by applying the combination treatment on healthy, nonwounded skin regions present on the combination-treated mice. This healthy region of the skin would receive the same 2 treatments as the wound site, and during tissue harvesting, this region was excised and preserved in 10% buffered formalin alongside a collection of unwounded skin samples from the untreated mice. Formalin-fixed samples were submitted to the Boston University Experimental Pathology Laboratory Service Core for histology processing and H&E staining. Histology slides were then visualized under an inverted microscope through a 60× objective.

### Statistics.

Statistical analysis was conducted through Student’s 2-tailed unpaired *t* test (2 groups) and 1-way ANOVA (3 or more groups). ***P < 0.001. **P < 0.01. *P < 0.05. NS, no significance (*P* > 0.05).

### Study approval.

Mouse abrasion model experimentation was approved by the Boston University Animal Care and Use Committee and was in accordance with NIH guidelines.

## Author contributions

PTD and JXC conceived the synergistic therapeutic treatment between photoinactivation of catalase and H_2_O_2_ or certain antibiotics. PTD, Y Zhu, and JH discovered that catalase from catalase-positive bacteria could be ubiquitously inactivated by 410 nm. PTD conducted catalase photoinactivation and intracellular bacteria assay. PTD, SJ, and JH conducted the in vitro CFU assay. JH conducted the biofilms experiment. PTD, SJ, and Y Zhan conducted the in vivo mouse abrasion experiments and histology assay. PTD and JXC cowrote the manuscript. GYL provided constructive suggestions for the project and manuscript. All authors read and commented on the manuscript.

## Supplementary Material

Supplemental data

Supplemental video 1

Supplemental video 2

## Version 1 04/21/2022

In-Press Preview

## Version 2 05/23/2022

Electronic publication
