# Supplementary material for: Photoinactivation of catalase sensitizes a wide range of bacteria to ROS-producing agents and immune cells
Source: JCI Insight. 2022 May 23;7(10):e153079. doi: 10.1172/jci.insight.153079 (PMC9220836; doi:10.1172/jci.insight.153079)
Supplement: Supplemental data [file jciinsight-7-153079-s084.pdf]

Supplementary information for manuscript

Photoinactivation of Catalase Sensitizes Wide-Ranging Bacteria to ROS-Producing Agents and Immune Cells

Pu-Ting Dong[1,2,#], Sebastian Jusuf[1,2,#], Jie Hui[1,2,#], Yuewei Zhan[1,2,#], Yifan Zhu[2,3], George Y. Liu[4,5], Ji-Xin Cheng[1,2,3,*]

[1]Department of Biomedical Engineering, Boston University, Boston, MA02215, USA.

[2]Photonics Center, Boston University, Boston, MA02215, USA.

[3]Department of Chemistry, Boston University, Boston, MA02215, USA.

[4]Division of Pediatric Infectious Diseases and Research Division of Immunology, Department of Biomedical Sciences, Cedars-Sinai Medical Center, Los Angeles, California, USA.

[5]Division of Infectious Diseases, Department of Pediatrics, UCSD, San Diego, California, USA.

*Corresponding author. Email: jxcheng@bu.edul; Phone: (617)-353-1276.

[#]These authors contributed equally: Pu-Ting Dong, Sebastian Jusuf, Jie Hui, Yuewei Zhan.

This file includes:

Supplementary Figures 1-6

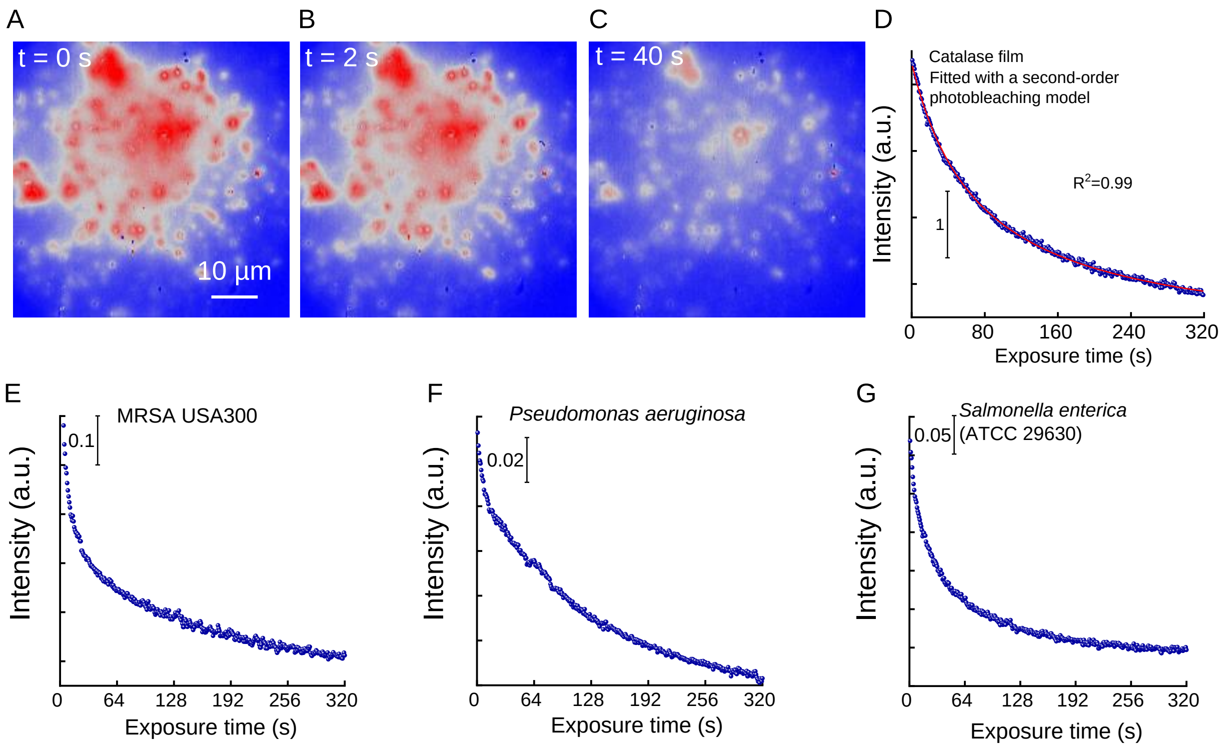

Supplementary Figure 1. Characterization of photoinactivation of catalase under transient absorption microscope. **A-C**. Transient absorption images of bovine liver catalase dried on a cover slide under different exposure time. **D**. Time-lapse transient absorption signals of dried bovine liver catalase. Curve fitted by a second-order photobleaching model. **E-G**. Time-lapse transient absorption signals of dried MRSA USA300 (**E**), *P. aeruginosa* (**F**), and *Salmonella enterica* (**G**). Pump=410 nm, 5 mW on the sample; probe=520 nm, 7 mW on the sample. Scalar bar=10 µm.

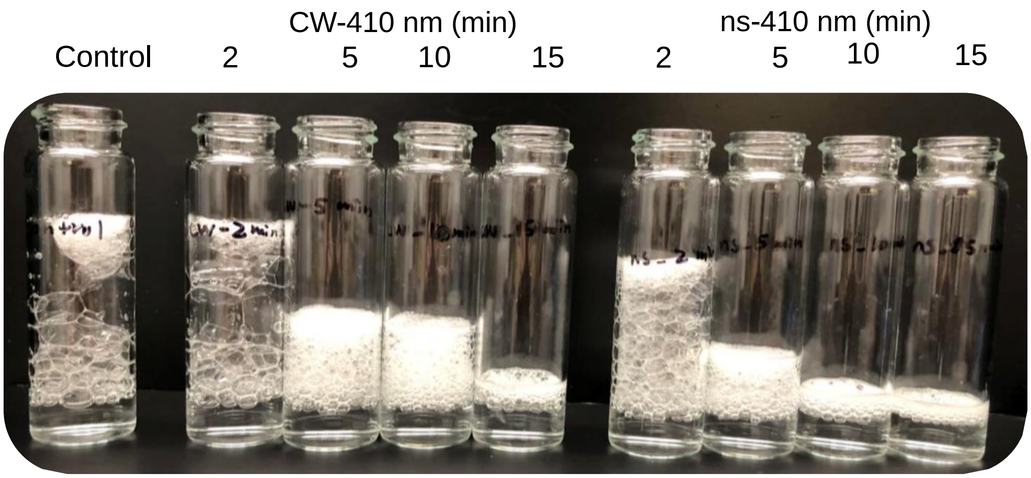

Supplementary Figure 2. Bubble test comparison between CW-410 nm and ns-410 nm exposure on the capability to inactivate bovine liver catalase. 410 nm: 50 mW/cm^2.

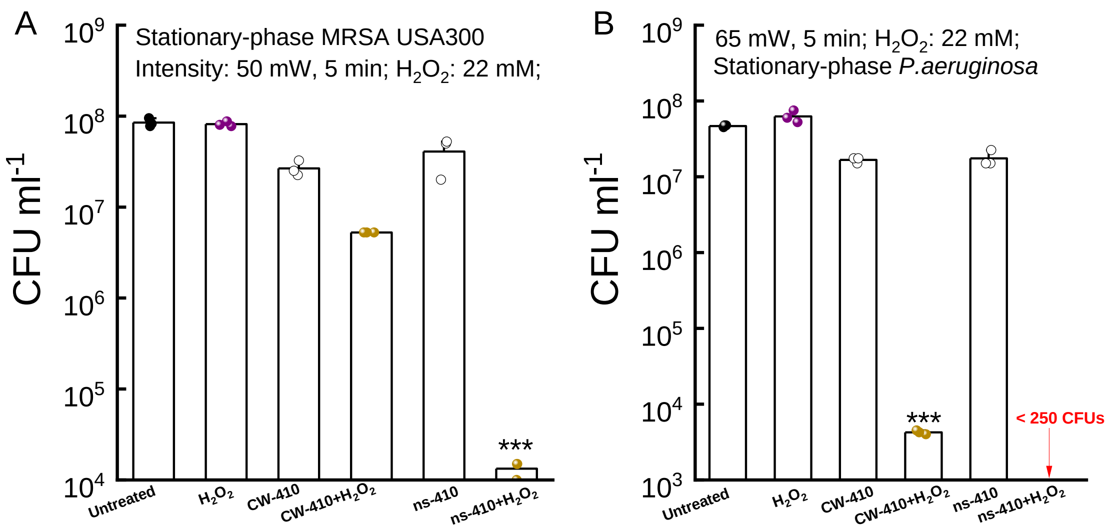

Supplementary Figure 3. CFU ml^{-1} of stationary-phase MRSA USA300 (A) and *P. aeruginosa* PAO1 (B) under CW-410 nm and ns-410 nm treatments. H_2O_2: 30-min incubation time. Data: Mean+SD. *N*=3. Student unpaired *t*-test. ***: $p<0.001$.

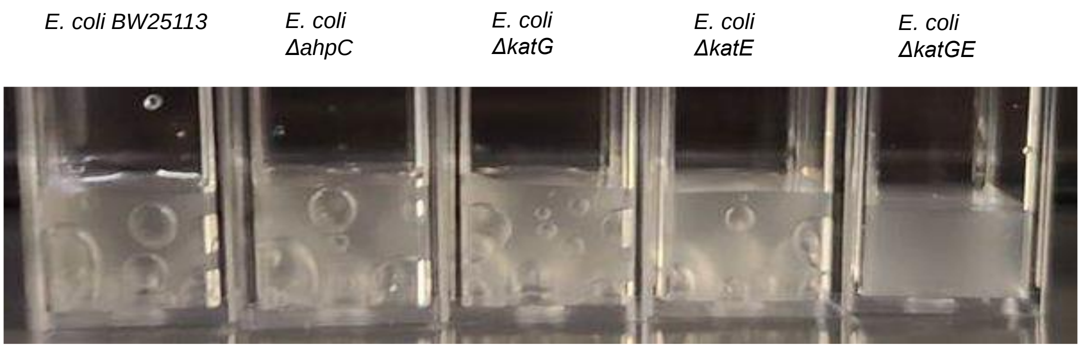

Supplementary Figure 4. Bubble formation of different *E. coli* strains in the presence of 3% H_2O_2.

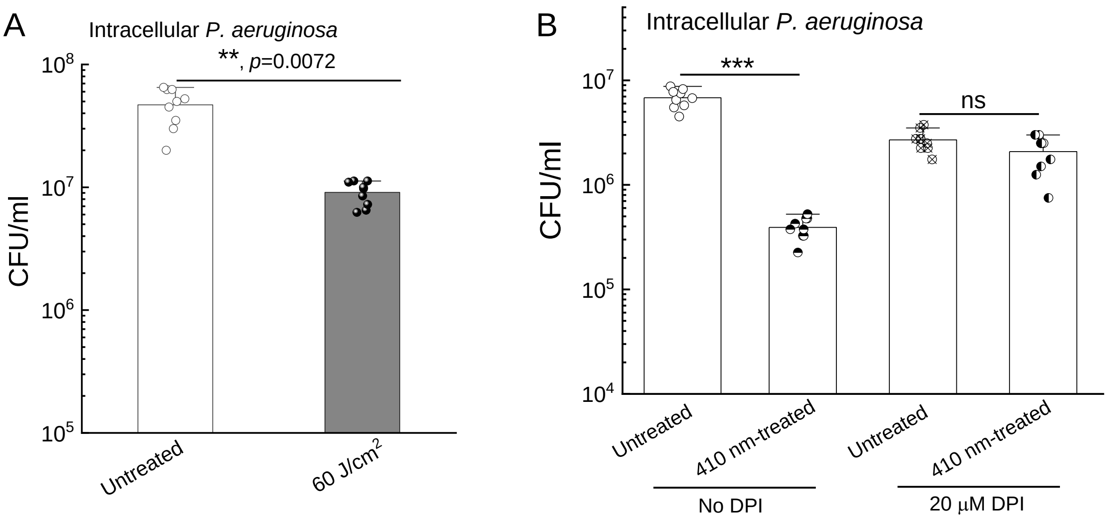

Supplementary Figure 5. CFU/ml of intracellular *P. aeruginosa* under different treatment schemes. **A**. CFU/ml of *P*. *aeruginosa* after *P*. *aeruginosa* infected macrophages with/without 410 nm blue light treatment. Dose: 60 J/cm^2. **B**. CFU/ml of *P*. *aeruginosa* after untreated and 410 nm-treated *P*. *aeruginosa* infected macrophages for two hours in the absence/presence of DPI (NOX2 inhibitor). Data: Mean+SD from at least three biological replicates (each biological replicate contains three technical replicates). Significant difference was determined through student unpaired *t*-test. **: $p<0.01$. $N=3$.

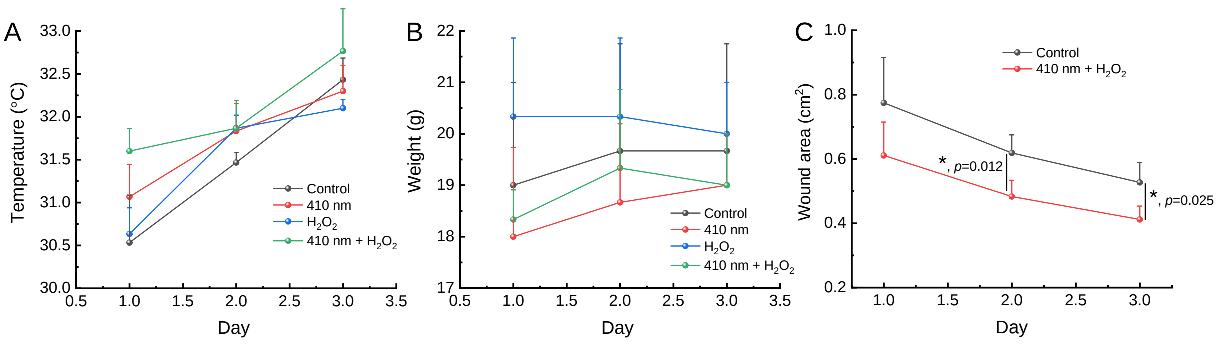

Supplementary Figure 6. Characterization of mouse physiology in terms of body temperature (A), weight (B), and wound size (C) under different treatment schemes. Dose: 60 J/cm^2. H_2O_2: 0.5%. Data: Mean+SD from at least three biological replicates. Significant difference was determined through the student unpaired *t*-test. *: p<0.05.
